# The prevalence of psychiatric disorders among students aged 6~ 16 years old in central Hunan, China

**DOI:** 10.1186/s12888-018-1823-7

**Published:** 2018-07-28

**Authors:** Yan-Mei Shen, Bella Siu Man Chan, Jian-Bo Liu, Yuan-Yue Zhou, Xi-Long Cui, Yu-Qiong He, Yu-min Fang, Yu-Tao Xiang, Xue-Rong Luo

**Affiliations:** 1 Department of Psychiatry, The Second Xiangya Hospital, Central South University, Changsha, 410011 Hunan China; 2 Mental Health Institute of Central South University, China National Clinical Research Center on Mental Disorders (Xiangya), China National Technology Institute on Mental Disorders, Hunan Technology Institute of Psychiatry, Hunan Key Laboratory of Psychiatry and Mental Health, Changsha, 410011 Hunan China; 3 The Department of Educational and Counselling Psychology, and Special Education, The University of British Columbia, Vancouver, Canada; 4 Mental Health Zhejiang University School of Medicine Hangzhou Seventh People’s Hospital, Hangzhou, 310013 China; 5 Unit of Psychiatry, Faculty of Health Sciences, University of Macau, Macao SAR, China

**Keywords:** Psychiatric disorder, Prevalence, Comorbidity, Gender, Epidemiology, ADHD

## Abstract

**Background:**

Though several epidemiological surveys of psychiatric disorders have been carried out in China, only a few of them are concerned about the prevalence of psychiatric disorders in central Hunan and reveal the distribution of common psychiatric disorders and their comorbidities.

**Methods:**

Achenbach’s Child Behavior Checklist (CBCL), the Mini International Neuropsychiatric Interview for Children and Adolescents (MINI-KID), and Diagnostic and Statistical Manual of Mental Disorders, 4th Edition (DSM-IV) were administered to a stratified sample of 17,071 participants aged 6 to 16 years old from two cities in the central part of Hunan province. Twelve-month prevalence rates were calculated.

**Results:**

Twelve-month prevalence of the population was 9.74%. The most common psychiatric disorders were attention deficit hyperactivity disorder (ADHD) (4.96%), oppositional defiant disorder (ODD) (2.98%) and generalized anxiety disorder (GAD) (1.77%). Of those with a 12-month prevalence diagnosis, 34.6% had one or more comorbid psychiatric disorders. Most notably, ADHD had comorbidity rates of 25.15% with ODD, 18.18% with CD, 6.38% with GAD, and 3.66% with MDD.

**Conclusions:**

Psychiatric disorders are common in Chinese children and adolescents. Being the most prevalent mental disorder, ADHD requires continued focus and support in awareness and education.

**Electronic supplementary material:**

The online version of this article (10.1186/s12888-018-1823-7) contains supplementary material, which is available to authorized users.

## Background

Studies focusing on mental disorders of children and adolescents have increased in recent decades [1]. The effect resulting from a psychological disorder not only impacts their quality of life but also predicts a higher chance of developing psychiatric disorders as they enter adulthood [2]. Children and youth with psychiatric disorders are six times more likely to experience health, legal, financial, and social problems as adults [3]. Numerous studies have indicated that children currently in treatment are less than the number of children with mental disorders, and underdiagnosis and undertreatment are major public health problems around the world [4–6]. Despite the critical need for information on the mental health of children and youth, research is scarce. The prevalence of mental health disorders among children and youth varies greatly in different parts of the world, ranging from 3.5 to 40.3% [7, 8]. This wide variation indicates great heterogeneity, which might result in a disparity of study goals, selection of study population, and criteria used to confirm the diagnoses of disorders [9]. China has a population of 1.3 billion, of which 238 million are children under 15 years of age [10]). Research on the prevalence of mental health disorders in children and youth across China is sorely lacking. Of the few epidemiological studies, they were all conducted in Hunan, Liaoning, and Sichuan provinces, which had a prevalence of 16.22% [11] (2005), 15.24% (2015) [12], and 9.15%(2007) [13], respectively. In the recent 10 years of China, the gross domestic product (GDP) had increased drastically from 18,731.89 billion to 68,905.21 billion RMB. The GDP of Hunan province is 2890.221 billion RMB, which is four times more than that in 2005 (659.61 billion RMB). The huge change suggested that an update in the prevalence rate of mental disability in health care is necessary. The purpose of this study is to investigate the prevalence, distribution, and comorbidities of common psychiatric disorders after 10 years of economic growth. Up-to-date prevalence estimates are vital for service delivery, resource provision, and research developments [14]. Likewise, identifying accurate prevalence variability can help to address queries about etiology and advise the design of future studies.

## Methods

### Samples and procedure

**Fig. 1:**
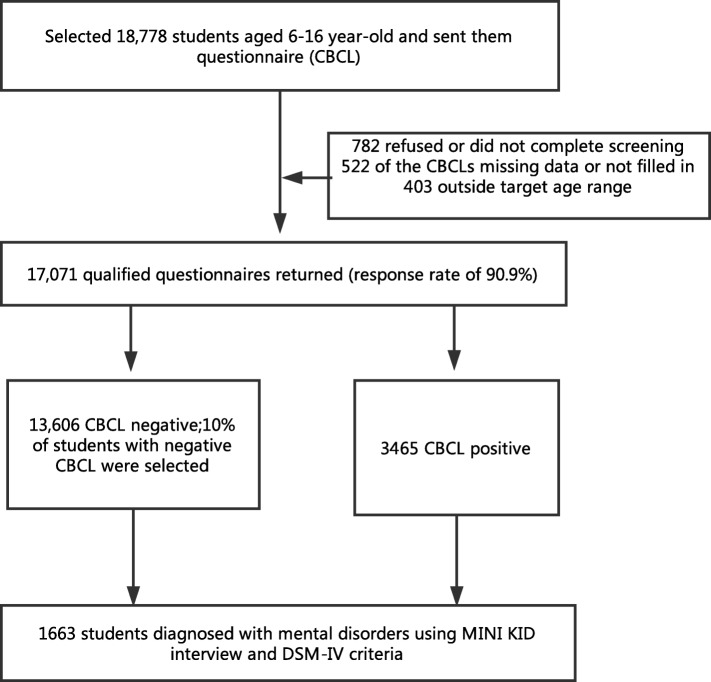
Procedure of Investigation

Hunan province is located in South China, with an area of 21.18 million square kilometers, accounting for 2.2% of China’s land area. It consists of 13 cities and 1 autonomous prefecture, with an overall population of 67.8 million in 2015. Two cities located in the central part of Hunan province, Changsha (population of 7 million) and Yiyang (population of 4.8 million), were selected for the study. The sample size of this study was based on the sample size of the national investigation of psychiatric disorder in China held by Beijing Anding Hospital, which was originally supposed to be 14,000. However, our study included 17,000 participants to ensure that we can obtain an adequate number in case of participation refusal. Thirteen schools, including two urban middle schools, four urban primary schools, three rural middle schools, and four rural primary schools, were selected at random. Approximately 18,778 students were enrolled, of which 17,071 participants (90.9%) gave consent to participate. Of the eligible participants, 782 refused or did not complete their questionnaire, and of those who completed, 522 contained missing data (over 10% incomplete on the questionnaire), and 403 were outside the age range. This was a two-stage study that used Achenbach’s Child Behavior Checklist (CBCL) as a screening tool, the Mini International Neuropsychiatric Interview for Children and Adolescents (MINI-KID), and the DSM-IV criteria to establish the final diagnosis. Questionnaires were sent to the students, and they brought the scale home for their parents to fill. Of the 17,071 qualified participants who completed the questionnaires, 13,606 participants scored negative to diagnosis, and 3465 scored positive, hinting a potential diagnosis of a disorder. Ten percent of the students with negative scores were selected randomly as controls. All of the 3465 participants with positive scores were given a full diagnostic interview using the MINI-KID (Sheehan et al., 2010) (parent and child versions). For those participants whose parents were unable to perform the diagnostic interview, the teachers were interviewed as alternative informants. Each interview lasted 15–40 min. For any participant with at least one diagnosis or a suspected diagnosis – based on either the parent or the child version of the MINI-KID – a diagnosis would be confirmed using the DSM-IV criteria. Through this procedure, 1663 participants were verified as having a diagnosis of at least one mental health disorder (see Fig. 1).

Eligible subjects were invited to participate in this study based on the following criteria: 1) enrolled students aged 6–16 years; 2) whose parents or guardians have given consent to participate; and 3) who had been living in the investigated sites for at least six months with household municipal registration. Exclusion criteria included the following: 1) informed consent not obtained, 2) students in special schools or not attending school, or 3) students could not be located after at least three attempts to visit at different times. The research protocol was approved by the Ethics Committee of the Second Xiangya Hospital, Central South University, China.

### Screening tool

The CBCL is a parent−/caregiver-completed questionnaire that assesses behavioral problems, emotional difficulties, and social competencies of a child. This standardized and objective measurement tool has been revised and widely used by child psychiatrists, pediatricians, developmental psychologists, and other mental health professionals for clinical and research purposes [15]. The Chinese version of CBCL (1991) has demonstrated to have high psychometric properties as a screening tool [16]. The CBCL has 113 items, providing scores for three broad-band scales: internalizing (sum of withdrawn, somatic complaints, and anxious/depressed subscales), externalizing (sum of attention problems and aggressive and delinquent behavior subscales), and total behavior problems. The subscales differ depending on age and gender. Each item is scored on a three-point Likert scale (0 = not true, 1 = somewhat or sometimes true, or 2 = very true or often true). Participants were asked to choose the scale point that best described their child’s behavior in the preceding six months [17]. The threshold value of the CBCL used in this study was based on the model for Chinese children and adolescents, and participants were considered to be screened positive when their score meet the criteria of each subscale [18] (see Additional file 1).

### Diagnostic criteria and tools

The MINI-KID is a guided structured interview that assesses psychiatric disorders based on the DSM-IV and ICD-10 in children and adolescents aged 6 to 17 years in a comprehensive and concise way. Both the parent and child participated in the interview, although it can also be administered solely to the children themselves [19]. The MINI-KID, which follows the structure and format of the adult version of the interview (MINI), was designed based on the Structured Clinical Interview for DSM-III-R [20] and the World Health Organization-designed Composite International Diagnostic Interview [21]. The assessment tool is divided into diagnostic sections or modules. It uses two to four questions that screen the respondent for each corresponding disorder. Additional questions of each disorder in a yes or no format will be used further if screened questions are positive. In response to discrepancies, further information will be obtained and concluded through clinical judgement. At the end of the assessment, a summary of the diagnostic criteria will be provided based on each disorder section. The instrument also screens for 24 DSM-IV and ICD-10 psychiatric disorders and suicidality of the target individual. Research had shown that the use of standardized structured and semi-structured diagnostic interviews reduce the risk of inadequate assessment in children and adolescents [22–24]. One down side of these previously used diagnostic instruments is being time consuming and lengthy to complete; each diagnosis requires 2 to 3 h to administer [25]. The MINI-KID is a reasonably accurate and reliable tool that greatly reduces the length and complexity of administration that serves the same psychometric property of the original form [19]. Our study used the validated Chinese version of the MINI-KID. Both the parent and child versions of the Chinese version of the MINI-KID had been proven to be reliable and valid [26, 27]. Potential diagnoses were considered present when they met the criteria on either the parent or child version. The diagnosis established by the MINI-KID was verified by trained psychiatrists following the DSM-IV criteria.

### Statistical analysis

Data analyses were performed using SPSS version 18. To estimate the prevalence rate of mental health disorders and comorbidity, frequencies and 95% confidence intervals were calculated. Differences in the prevalence of mental health disorders between different genders and age groups were assessed using the Chi-square (*χ*^2^) test. All statistical tests were two tailed, with 0.05 as the significance level.

### Quality control

#### Quality control for the MINI-KID

Eighteen psychiatrists and 10 graduate students had 6 h of MINI-KID administration training. Two children and parents were interviewed using the MINI-KID, and consistency checks were conducted after the training, with a Kappa of 0.774.

#### Quality control for the diagnoses

Twelve registered psychiatrists received 8 h of training using the DSM-IV criteria for mental health disorders in children and youth. Two patients were interviewed for the 24 psychiatric disorders using the MINI-KID, and consistency check was conducted after the training, with a Kappa between 0.77 and 0.91.

## Results

This study comprised of 4468 urban and 12,603 rural students, with a gender distribution of 8841 males and 8230 females. The 12-month prevalence of mental health disorders was 9.74%. The prevalence (11.79%) in boys was significantly higher than that (7.55%) in girls (*P* < 0.05; Table 1). The prevalence rate was 9.33% in ages under 11 years, 10.11% in 12–14 years, and 9.98% above 15 years (*P* > 0.05; Table 1). The prevalence difference between rural and urban areas (9.70% vs 9.85%) was not significant (*P* > 0.05; Table 1).

**Table 1:** Twelve-month Prevalence, gender, age and urban-rural distribution of mental disorders among children and adolescents

Variable	Sample Rate (%)	95% CI	χ^2^	*P*
Prevalence	17,071(9.74%)	9.30~ 10.18%		
Gender				
Boys	8841(11.79%)	11.12~ 12.46%	87.165	<0.001
Girls	8230(7.55%)	6.98~ 8.12%		
Age				
≤ 11 year	7689(9.33%)	8.68~ 9.98%	2.796	0.247
12–14 year	7198(10.11%)	9.41~ 10.81%		
≥ 15 year	2184(9.98%)	8.72~ 11.24%		
Community				
Rural	12,603(9.70%)	9.18~ 10.22%	0.078	0.781
Urban	4468(9.85%)	8.98~ 10.72%		

Attention deficit hyperactivity disorder (ADHD) ranked the highest with a prevalence of 4.96%, followed by oppositional defiant disorder (ODD) at 2.98%, generalized anxiety disorder (GAD) at 1.77%, and conduct disorder (CD) at 1.39%. Boys showed a significantly higher prevalence than girls in terms of ADHD, ODD, and CD, whereas girls showed a higher prevalence in terms of GAD and major depressive disorder (MDD). Participants aged ≤11 years showed the highest prevalence in ADHD, CD, and ODD (see Table 2).

**Table 2:** Prevalence of common disorders and their distribution among gender, different age group and district

Disorders	Total N(%)	95%CI	Boys N(%)	Girls N(%)	≤11 year N(%)	12–14 year N(%)	≥15 year N(%)	Rural N(%)	Urban N(%)
ADHD	847(4.96)	4.64%--5.29%	628(7.1)	219(2.66)	438(5.7)	331(4.6)	78(3.57)	623(4.94)	224(5.01)
ODD	508(2.98)	2.72%--3.23%	309(3.5)	199(2.42)	240(3.12)	212(2.95)	56(2.56)	378(3.00)	130(2.91)
GAD	302(1.77)	1.57%--1.97%	114(1.29)	188(2.28)	65(0.85)	169(2.35)	68(3.11)	216(1.71)	86(1.92)
CD	237(1.39)	1.21%--1.56%	187(2.12)	50(0.61)	110(1.43)	93(1.29)	34(1.56)	193(1.53)	44(0.98)
SPP	126(0.74)	0.61%--0.87%	59(0.67)	67(0.81)	35(0.46)	60(0.83)	31(1.42)	100(0.79)	26(0.58)
TD	105(0.62)	0.50%--0.73%	43(0.49)	62(0.75)	23(0.3)	65(0.9)	17(0.78)	75(0.60)	30(0.67)
MDD	104(0.61)	0.49%--0.73%	30(0.34)	74(0.9)	17(0.22)	71(0.99)	16(0.73)	75(0.60)	29(0.65)
OCD	98(0.57)	0.46%--0.69%	45(0.51)	53(0.64)	25(0.33)	53(0.74)	20(0.92)	69(0.55)	29(0.65)
Dysthymia	29(0.17)	0.11%--0.23%	9(0.1)	20(0.24)	7(0.09)	19(0.26)	3(0.14)	22(0.17)	7(0.16)
Mania	25 (0.15)	0.10%--0.22%	22 (0.25)	3 (0.04)	13 (0.17)	9(0.13)	3(0.14)	14(0.11)	11(0.25)
SOP	12(0.07)	0.03%--0.11%	6(0.07)	6(0.07)	4(0.05)	7(0.1)	1(0.05)	10(0.08)	2(0.04)
PDD	12(0.07)	0.03%--0.11%	8 (0.09)	4 (0.05)	5 (0.07)	5(0.07)	2(0.09)	9(0.07)	3(0.07)
PTSD	4(0.02)	0.00%--0.05%	1(0.01)	3(0.04)	2(0.03)	0(0)	2(0.09)	4(0.03)	0(0)
SAD	3(0.02)	0.00%--0.04%	0(0)	3(0.04)	3(0.04)	0(0)	0(0)	2(0.02)	1(0.02)

*ADHD* Attention deficit hyperactive disorder, *ODD* oppositional defiant disorder, *CD* conduct disorder, *GAD* generalized anxiety disorder, *PTSD* post-traumatic stress disorder, *OCD* obsessive compulsive disorder, *SOP* social phobia, *SPP* specific phobia, *SAD* separation anxiety disorder, *MDD* major depressive disorder, *TD* tic disorder, *PDD* pervasive developmental disorders

Nearly one third of the participants showed at least one psychiatric comorbidity; girls and those aged 12–16 years showed a higher proportion of comorbidity than the other groups (see Table 3). ADHD is most frequently comorbid with ODD (25.15%), CD (18.18%), and GAD (6.38%). In addition, GAD is most likely comorbid with ADHD (17.88%), ODD (17.88%), and MDD (16.56%), whereas MDD with GAD (48.08%) and tic disorder (47.12%) (see Table 4).

**Table 3:** Distribution of comorbidities among different gender, age group and district

	Diagnosed with only 1 kind of Disorder	Comorbid with 1 kind of disorder	Comorbid with 2 kinds of disorder	Comorbid with 3 or more kinds of disorder	χ^2^	*p*
Total	1087(65.4%)	391(23.5%)	123(7.4%)	62(3.7%)		
Boys	699(67.1%)	263(25.2%)	57(5.5%)	23(2.2%)	36.11	< 0.001
Girls	388(62.5%)	128(20.6%)	66(10.6%)	39(6.3%)		
6–11 years old	503(68.5%)	182(24.8%)	39(5.3%)	10(1.4%)	30.37	< 0.001
12–16 years old	584(62.9%)	209(22.5%)	84(9.0%)	52(5.6%)		
Urban	293(66.6%)	100(22.7%)	31(7.0%)	16(3.6%)	0.408	0.939
Rural	794(64.9%)	291(23.8%)	92(7.5%)	46(3.8%)

**Table 4:** Comorbidity of several common mental disorders with other kinds of common DSM-IV mental disorders

	ADHD(%)	ODD(%)	GAD(%)	CD(%)	SPP(%)	TD(%)	MDD(%)	OCD(%)	Dysthymia(%)	Mania(%)
ADHD	/	213(25.15)	54(6.38)	154(18.18)	26(3.07)	14(1.65)	31(3.66)	27(3.19)	3(0.35)	2(0.24)
ODD	213(41.93)	/	54(10.63)	/	16(3.15)	9(1.77)	12(2.36)	23(4.53)	3(0.59)	2(0.39)
GAD	54(17.88)	54(17.88)	/	23(7.62)	22(7.28)	38(12.58)	50(16.56)	1(0.33)	12(3.97)	/
CD	154(64.98)	/	23(9.7)	/	15(6.33)	5(2.11)	19(8.02)	14(5.91)	2(0.84)	1(0.42)
SPP	26(20.63)	16(12.7)	22(17.46)	15(11.9)	/	7(5.56)	12(9.52)	6(4.76)	9(7.14)	/
TD	14(13.33)	9(8.57)	38(36.19)	5(4.76)	7(6.67)	/	49(46.67)	10(9.52)	20(19.05)	/
MDD	31(29.81)	12(11.54)	50(48.08)	19(18.27)	12(11.54)	49(47.12)	/	15(14.42)	/	/
OCD	27(27.55)	23(23.47)	1(1.02)	14(14.29)	6(6.12)	10(10.2)	15(15.31)	/	7(7.14)	/

## Discussion

### Prevalence

The 12-month prevalence of any type of psychiatric disorders (9.74%) was within the range of 3.5–40.3% as reported previously [7, 8]. The prevalence in this study was higher than that in Ethiopia (3.5%) [7], Italy (8.2%) [28], Norway (7.0%) [29], and Malaysia (6.1%) [30], but similar to that in Mexico (9.1%) [31], Northeast China (9.49%) [32], and India (12.5%) [33] and lower than that in Russia (15.3%) [34], the Netherlands (22.0%) [35], Ireland (27.4%) [36], America (21.6%) [37], and Chile (38.3%) [38]. The discrepancy between studies may have been caused by several factors. First, various screening and diagnostic tools and different versions of the same instrument used contribute to the fluctuation in the prevalence rate [39]. For instance, a survey conducted in the United Arab Emirates found a prevalence of 15.6% using the Self-Reporting Questionnaire but reported a prevalence of 8.2% using the Composite International Diagnostic Interview [40]. Second, sampling methods may result in inconsistent rates. A meta-analysis reported that prevalence rate following no requirement of impairment was 27.1%, whereas it was 14.0% when disorder-specific impairment was defined by the diagnostic interview [9]. This survey was a standard two-stage study involving the criteria of social impairment, which may lead to low prevalence rate. Third, as much stigma is associated with mental illness in traditional Chinese culture, many Chinese patients tend to deny mental disorders [41]. Many Chinese patients will either avoid seeking medical help for mental health issues or may only bring forward complaints of somatic symptoms. All these factors can result in under- or misdiagnosis [42].

The prevalence of psychiatric disorder was lower than the pooled prevalence of 13.4%. The prevalence of GAD, MDD, and CD was also lower than the pooled prevalence of each kind of disorder reported in a meta-analysis, which indicated a prevalence of 6.5% (anxiety disorder), 2.6% (depressive disorder), and 5.7% (disruptive disorder). By contrast, ADHD was higher than the pooled prevalence of ADHD. One explanation for this perhaps would be that the prevalence rate reported in the meta-analysis study included all types of depressive and anxiety symptoms, whereas our study reported on the prevalence of GAD and MDD. Another reason would be the pressure from public discrimination and stigmatization because Chinese individuals feel ashamed to disclose affective symptoms [43]. Furthermore, our study reported higher prevalence rate than the pooled prevalence rate. One reason for this discrepancy might be that parents often consider mild to moderate intensity of oppositional and problem behaviors as symptoms of ADHD, whereas moderate to severe behaviors are considered to be ODD and CD [44].

The prevalence of psychiatric disorders is 9.7% among children and adolescents in our study was lower than the prevalence reported in 2005. There are several reasons contributing to this phenomenon. Firstly, the diagnostic tool is different; in 2005, the diagnostic tool used was Kiddie Schedule for Affective Disorders and Schizophrenia Present and Lifetime Version (K-SADS-PL), and in this study, we used MINI-KID as the diagnostic tool. Secondly, the prevalence of 16.2% reported in 2005 included more psychiatric disorders than this study. In addition to the psychiatric disorders included in MINI-KID, their study also included acute stress disorder, communication disorder (including expressive language disorder, phonological disorder, stutter, and mixed receptive-expressive language disorder), sleep disorders (including dyssomnia, nightmare disorder, sleep terror disorder, and sleep walking disorder), elimination disorders (including encopresis and enuresis), and elective mutism. This broader spectrum of disorders may have resulted in a higher prevalence. Thirdly, though psychiatric disorders remained prevalent, given the rapid economic growth in China, mental health services were improved [45], and several studies have reported that mental health problems have been negatively associated with economic growth in China [46, 47].

### Gender difference

The current study shows significant gender differences, i.e., prevalence of ADHD, ODD, and CD in boys was higher than that in girls, which is consistent with other studies [11, 48]. Various theories were associated with gender differences. First, the current diagnostic criteria could not fully cover all relevant symptoms in female patients. For example, one study [49] found that a group of untreated girls who did not meet the DSM criteria presented pronounced ODD symptoms and were almost as functionally impaired as girls who met the diagnostic criteria. Interestingly, no boys were found to meet the functional impairment criteria using the same method. This finding suggested the potential underdiagnoses in girls. Second, as symptoms in boys tend to be external problem behaviors, such as hyperactivity, compulsivity, and aggressive behaviors, they are easy to identify. Girls, however, were more likely to exhibit subtle internal problem behaviors, which were more likely to be overlooked [50]. Furthermore, previous studies found biological factors associated with the gender differences in the prevalence of disruptive behavior disorders and externalizing problems [51]. Specifically, Y-linked variants play a role in impulsivity and aggression in boys with ADHD [52]. Approximately half of the variance related to the development of externalizing behaviors are accounted for by biological factors [53].

We also found that girls tend to suffer from GAD and MDD, which is consistent with previous findings [54]. When confronted with stressors, females are likely to be negatively affected [55]. This gender-specific risk might be caused by the inability to regulate negative emotional responses of stress and fear [56]. Generally, females and males confront similar stressors differently, with females being more vulnerable to developing depression and related anxiety disorders than males [57]). Moreover, the hypothalamic–pituitary–adrenal (HPA) axis affects the levels of various hormones, including cortisol. Individuals with mood disorders often show elevated cortisol responses to stress, indicating dysregulation in the HPA. Ovarian hormones found in females also modulate HPA regulation [58]. Symptoms of emotional disorders may be more predominant during rapid changes in levels of ovarian hormones, such as in puberty, as hormonal fluctuation triggers dysregulation of the stress response.

### Comorbidity

Our study found that ADHD is the most common comorbid disorder, which is consistent with previous findings [59]. Our study showed that ADHD patients have comorbidity rates of 48.2% with at least one psychiatric disorder, 36.4% with one psychiatric disorder, 8.5% with two kinds of psychiatric disorders, and 3.3% with three or more kinds of psychiatric disorders, which was similar to the rate of 52% reported in Denmark in a large sample [60] and lower than the rate of 66% reported in Italy [61]. Jensen and Steinhausen suggested that a smaller sample size and the absence of an assessment of impaired functioning may have contributed to a higher comorbidity rate [60]. Contrastingly, both our study and the study conducted in Denmark [60] had large samples and included an assessment of impaired functioning, and thus resulted in lower comorbidity rates. Also, the comorbidity of ADHD is considered to be developmental and dynamic [60]; because our study was a cross-sectional study, the participants might have not yet developed a comorbid psychiatric disorder when they were investigated, which might also have resulted in a lower comorbidity rate being presented. The mechanism behind the high comorbidity rate of ADHD and psychiatric disorders remains unclear. Heidbreder (2015) has suggested that, because of the considerable overlapping symptoms between ADHD and its comorbid psychiatric disorders, ADHD should be considered as a spectrum disorder using a dimensional rather than categorical diagnosis [62]. Other studies have also suggested that ADHD and psychiatric disorders might share similar brain regions [63].

Disruptive behavior disorders including ODD and CD remain the most common comorbid disorders; our study reported a comorbidity rate of 25.15% with ODD, which lies in the range of 20~ 60% as reported by other studies [61, 64]**,** and a comorbidity rate of 18.8% with CD, which is slightly lower than the rate of 20% reported by Biederman [64], but higher than the rate of 16.5% reported by Jensen and Steinhausen [60]. Several reasons might contribute to this high comorbidity rate between ADHD and disruptive disorders. First, the symptoms of ADHD and ODD/CD are often correlated and change concurrently over time, and perhaps may be different manifestations or intensities of a similar disorder [65]. Reiff and Stein [44] have proposed that mild to moderate intensity of oppositional behaviors is likely to be a component of ADHD, whereas moderate to severe intensity is more likely to represent ODD/CD. Changes in the symptoms of ADHD, ODD, and CD over time are all correlated [66, 67]. Second, comorbidity could be linked to genetic dispositions; ADHD and disruptive disorders may be a result of shared genetic liabilities and similar interactions between genes and the environment [68].

Our study reported a comorbidity rate of 12.64% in anxiety disorders (including GAD, SPP and OCD), which was higher than the rate reported in Denmark [60], and lies within the range of 15~ 35% reported by Pliszka et al. [69]. Also, our study found that the comorbidity rate of MDD (3.66%) in ADHD patients is lower than the rate of 5% reported in Italy [61], and higher than that reported in Denmark [60] The comorbidity rate of affective disorder varied substantially, which might be related to epidemiological method and the population enrolled in the study [70].

### Strengths and limitations

The strengths of this study included the use of the two-stage method, standardized diagnostic tool, and large sample size. However, this study has several limitations. First, only students enrolled in mainstream schools were included, and children in special schools or who did not go to school were not included. Thus, the findings cannot represent all children and adolescents. Second, we failed to record the number of children with a diagnosis from the MINI-KID but did not meet the criteria of DSM-IV. We considered the diagnosis only made from both the MINI-KID and the DSM-IV criteria. Third, the prevalence of pervasive developmental health disorder was relatively low due to the use of the MINI-KID. The MINI-KID consists of four brief questions on the child’s social skills, repetitive interests, and behaviors. Finally, autism spectrum disorder (ASD) is a significant mental health disorder that we did not examine in this study. Future studies should examine the prevalence and distribution of ASD.

## Conclusion

Our study revealed that nearly one tenth of Chinese children and adolescents suffer from at least one type of mental health disorder, with a higher prevalence in boys than in girls. Early diagnosis and intervention can help young children when they are most vulnerable to minimize or overcome mental health disorders as they grow older. Over the course of childhood, for those who have a diagnosis, caregivers should be aware of comorbid disorder(s) – either present or potential – and take preventive measures and perform early screening. Doing so can detect underlying or emerging disorders early and help to alleviate a difficult transition into adolescence and adulthood.

## Additional file

Additional file 1: Demarcation and total scores for subscales of behavior problem. The Additional file 1 provided information on the demarcation score for the various subscales of behavior problem and total score in adolescents aged 6–16 years old. The scores are presented in 4 separate tables that are divided by gender (boys and girls) and by age group (6–11, 12–16). (DOCX 16 kb)

## Abbreviations

ADHD: Attention deficit hyperactivity disorder
CBCL: Achenbach’s Child Behavior Checklist
CD: Conduct disorder
DSM-IV: Diagnostic and Statistical Manual of Mental Disorders, 4th Edition
GAD: Generalized anxiety disorder
MDD: Major depressive disorder
MINI-KID: The Mini International Neuropsychiatric Interview for Children and Adolescents
OCD: Obsessive compulsive disorder
ODD: Oppositional defiant disorder
PDD: Pervasive developmental disorders
PTSD: Post-traumatic stress disorder
SAD: Separation anxiety disorder
SOP: Social phobia
SPP: Specific phobia
TD: Tic disorder
