# Supplementary material for: The prevalence of psychiatric disorders among students aged 6~ 16 years old in central Hunan, China
Source: BMC Psychiatry. 2018 Jul 28;18:243. doi: 10.1186/s12888-018-1823-7 (PMC6064142; doi:10.1186/s12888-018-1823-7)
Supplement: Supplementary file 1 — Demarcation and total scores for subscales of behavior problem. The Additional file 1 provided information on the demarcation score for the various subscales of behavior problem and total score in adolescents aged 6–16 years old. The scores are presented in 4 separate tables that are divided by gender (boys and girls) and by age group (6–11, 12–16). (DOCX 16 kb) [file 12888_2018_1823_MOESM1_ESM.docx]

The threshold value of the CBCL used in this study was based on the model for Chinese children and adolescents. The subjects were considered positive when their score meets the demarcation score for each subscale (SU et al., 1995).

Table 1. Demarcation score for subscales of behavior problem and total score in boys aged 6–11 years old.

| Type | Schizoid | Depression | Social problem | Compulsivity | Somatic complaints | Withdrawal | Hyperactivity | Aggressivity | Delinquency | Total |
| --- | --- | --- | --- | --- | --- | --- | --- | --- | --- | --- |
| Demarcation | 5 | 9 | 5 | 8 | 6 | 5 | 10 | 19 | 7 | 42 |

Table 2. Demarcation score for subscales of behavior problem and total score in girls aged 6–11 years old.

| Type | Depression | Withdrawal | Somatic complaints | Schizoid and compulsivity | Hyperactivity | Sexual problems | Delinquency | Aggressivity | Brutality | Total |
| --- | --- | --- | --- | --- | --- | --- | --- | --- | --- | --- |
| Demarcation | 3 | 8 | 8 | 3 | 10 | 3 | 2 | 18 | 3 | 41 |

Table 3. Demarcation score for subscales of behavior problem and total score in boys aged 12–16 years old.

| Type | Somatic complaints | Schizoid | Social problem | Immature | Compulsivity | Hostility | Delinquency | Aggressivity | Hyperactivity | Total |
| --- | --- | --- | --- | --- | --- | --- | --- | --- | --- | --- |
| Demarcation | 10 | 7 | 14 | 5 | 5 | 10 | 8 | 18 | 9 | 38 |

Table 4. Demarcation score for subscales of behavior problem and total score in girls aged 12–16 years old.

| Type | Anxiety/compulsivity | Somatic complaints | Schizoid | Depression/withdrawal | Immature | Delinquency | Aggressivity | Brutality | Total |
| --- | --- | --- | --- | --- | --- | --- | --- | --- | --- |
| Demarcation | 17 | 7 | 3 | 12 | 11 | 11 | 7 | 4 | 37 |

**Reference：**

1. SU L, Li X, Luo X, Yang Z, Wan G: **The standardization of Achenbach child behavior checklist in hunan** *Journal of clinical psychosomatic diseases* 1995(2):66-72.
